# A novel strategy to achieve effective drug delivery: exploit cells as carrier combined with nanoparticles

**DOI:** 10.1080/10717544.2016.1230903

**Published:** 2017-02-03

**Authors:** Liang Pang, Chun Zhang, Jing Qin, Limei Han, Ruixiang Li, Chao Hong, Huining He, Jianxin Wang

**Affiliations:** 1 Key Laboratory of Smart Drug Delivery, Ministry of Education, Department of Pharmaceutics, School of Pharmacy, Fudan University, Shanghai, China and; 2 Tianjin Key Laboratory on Technologies Enabling Development of Clinical Therapeutics and Diagnostics, School of Pharmacy, Tianjin Medical University, Tianjin, China

**Keywords:** Red blood cell, leukocyte, stem cell, carrier, nanoparticle

## Abstract

Cell-mediated drug delivery systems employ specific cells as drug vehicles to deliver drugs to targeted sites. Therapeutics or imaging agents are loaded into these cells and then released in diseased sites. These specific cells mainly include red blood cells, leukocytes, stem cells and so on. The cell acts as a Trojan horse to transfer the drug from circulating blood to the diseased tissue. In such a system, these cells keep their original properties, which allow them to mimic the migration behavior of specific cells to carry drug to the targeted site after *in vivo* administration. This strategy elegantly combines the advantages of both carriers, i.e. the adjustability of nanoparticles (NPs) and the natural functions of active cells, which therefore provides a new perspective to challenge current obstacles in drug delivery. This review will describe a fundamental understanding of these cell-based drug delivery systems, and discuss the great potential of combinational application of cell carrier and NPs.

## Introduction

Most of the bioactive drugs are restricted in the clinical application due to their low solubility, short exposure and serious side effects on off-target tissues. Various nanotechnology has been developed to overcome these shortcomings (De Souza et al., 2010; Yamashita & Hashida, 2013; Zhang et al., 2013), but it seldom brought satisfactory therapeutic outcomes. A pioneering study emerged in the 1970s that successfully encapsulated β-glucosidase and β-galactosidase into red blood cells (RBCs) for the treatment of Gaucher’s disease laid the foundation for future research and opened new avenue for cell-mediated drug delivery (Ihler et al., 1973). It inspired us to take advantage of cell’s natural function to deliver drug into diseased tissues. This cell-based drug delivery system offers several advantages compared to current nanoparticle drug delivery system, which include improved drug efficacy, extended half-lives, sustained drug release, and limited immunogenicity and cytotoxicity.

Red blood cell, leukocyte and stem cell, have been studied in cell-based therapy due to their long circulation and specific tropism to diseased tissue (Muller et al., 2006; Porada & Almeida-Porada, 2010; Chen & Liu, 2012; Lameijer et al., 2013). In fact, leukocytes and stem cells have been used for tumor immunotherapy and tissue regeneration in clinical trials (Sharma et al., 2014; Behfar et al., 2014; Firor et al., 2015; Ikeda & Shiku, 2015). In recent decades, surprising results were developed by exploiting cells as vehicles combined with nanodrugs for therapy. It was found that nanoparticle loading in cells did not affect its migration, chemotaxic ability. In addition, exosomes, cell membrane components, microvesicles, which originated from cells, can mimic the function of cells to deliver drug into targeted tissue in noninvasive way (Haney et al., 2015; Hu et al., 2015; Peng et al., 2015).

This review provides an overview of recent updates of cell as carrier to deliver drugs into diseased sites. Particularly, we highlight three areas: 1. Cell choices for drug carrier, 2. The application of cell as carrier combined with NPs in disease therapy and 3. Construction, evaluation of “Cell-NPs” system.

## Nanoparticle drug delivery systems

Nanoparticle drug delivery systems carry multiple therapeutic agents such as small drugs, peptides and proteins, and recently plasmid DNA, and offer several distinct advantages over free drug (Gradauer et al., 2013; Wang et al., 2014; Samanta et al., 2015). Nanoparticles (NPs) can be adjusted to have specific physicochemical properties, including size, surface charge, hydrophobicity/hydrophilicity and shape, depending on their application (Albanese et al., 2012; Ma et al., 2013). Nanosystems modified with different biological compositions have been extensively investigated for drug delivery applications. The advantages of NPs can be summarized as below: (1) Drugs are loaded in, adsorbed or chemically coupled onto the NPs surface. In this way, NPs shell could protect therapeutic agents from degradation or deactivation prior to reaching target sites and allow their sustained release; (2) Coating the surface of NPs with polyethylene glycol (PEG), or “PEGylation”, is a commonly used approach for shielding NPs from aggregation, opsonization and phagocytosis, prolonging systemic circulation time (Sriraman et al., 2015; Suk et al., 2015; Deng et al., 2016); (3) NPs functionalized with target moieties are used for targeted delivery, which significantly improve the drug distribution in diseased tissues (Garg et al., 2015). (4) The compositions of stimulus-responsive polymers in NPs facilitate achieving a well-controlled drug release (Taghizadeh et al., 2015).

However, it has been shown that these techniques usually cannot completely prohibit protein opsonization and phagocytic activity of the reticular endothelial system (RES system). Targeted efficiency also struggles to meet our demands. In addition, the presence of anti-PEG antibodies may limit therapeutic efficacy of PEGylated substances as a consequence of inducing rapid clearance and decreasing biological activity of the substances by neutralization (Ishida & Kiwada, 2013).

## Cell choices for carriers and its application combined with NPs

### Red blood cells

**Figure 1.**
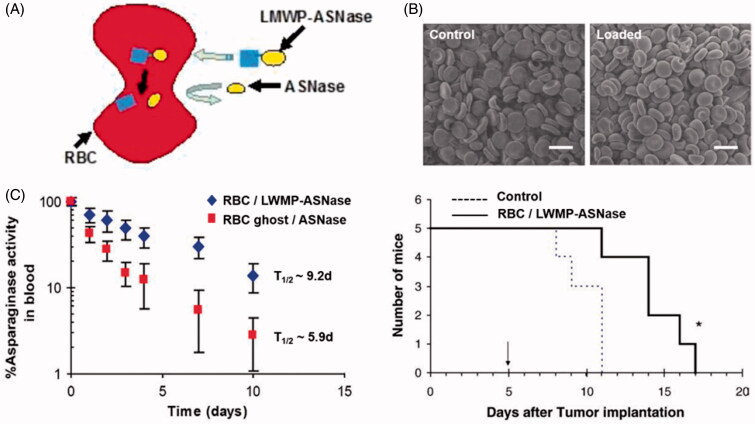
(A) Illustration of CPP mediated RBCs encapsulation technology (He et al., 2014). (B) The morphology of untreated normal RBCs and LMWP-ASNase-encapsulated RBCs by scanning electron microscopic images. (C) Pharmacokinetic profiles of ASNase in DBA2 mice and Kaplan–Meier survival curve for DBA/2 mice bearing L5178Y lymphoma cells (Kwon et al., 2009).

Of all types of cells in body, RBCs are the most readily available and abundant cells. They transport oxygen and nutrient to all parts of the body as natural carriers. They possess a number of favorable characteristics which make them interesting for constructing RBC-mediated drug delivery system (Bhateria et al., 2014). First, they have a long life-span of about 120 days and have a widespread circulation throughout body, and hence, can be used as drug reservoir enabling them to facilitate sustained drug release into the blood; second, they protect encapsulated drugs from degradation; and third, they are completely biodegradable without generating toxic products and show no or only minor immunogenic responses. A large number of studies tried to prolong the retention time of drugs in the circulation by anchoring them onto the surface of RBC membranes, or by loading it into the RBC. Hypotonic dialysis is widely used in loading drugs into RBCs, the encapsulation process involves reversible hypotonic swelling of the RBCs that transiently opens pores in the membrane, allowing drugs to diffuse into the cells. Upon return into isotonic medium, the pores are resealed and the drug is entrapped (Gutierrez Millan et al., 2004). In this approach, gold (Au) nanoparticles (AuNPs) were incorporated into RBCs to produce dynamic X-ray imaging of blood flows with high image contrast (Ahn et al., 2011). However, RBCs undergo a degree of structural and chemical composition changes during the loading procedure, these changes are recognized by RES organs and affect the normal function of RBCs, within a short time period after injection (Hamidi et al., 2007; Briones et al., 2009). Recently, a novel encapsulation method was introduced to overcome these obstacles, which called for covalent conjugation of the drug with cell-penetrating peptides (CPPs) via disulfide linkage (He et al., 2014). The CPP-conjugate was internalized into erythrocytes without altering RBCs’ structural and functional attributes. After entering into the erythrocyte, CPP was detached from drug via degradation of the disulfide linkage in the presence of a high level of cytoplasmic glutathione. As shown in Figure 1, the cell-penetrating low molecular weight protamine (LMWP) peptide was conjugated to l-asparaginase via a disulfide bond to treat acute lymphoblastic leukemia. The l-asparaginase-entrapped RBCs not only had resemblance to the profile of the native erythrocytes, but also produced a much enhanced therapeutic efficacy of the entrapped drug (Kwon et al., 2009). After intravenous injection into DBA/2 mice, as a control group, RBCs loaded with l-asparaginase via hypotonic methods had a half-life of approximately 5.9 days, while l-asparaginase-loaded erythrocytes with LMWP-mediated encapsulation yielded a prolonged plasma half-life of 9.2 days and significantly increased the survival time of mice.

### Leukocytes

**Figure 2.**
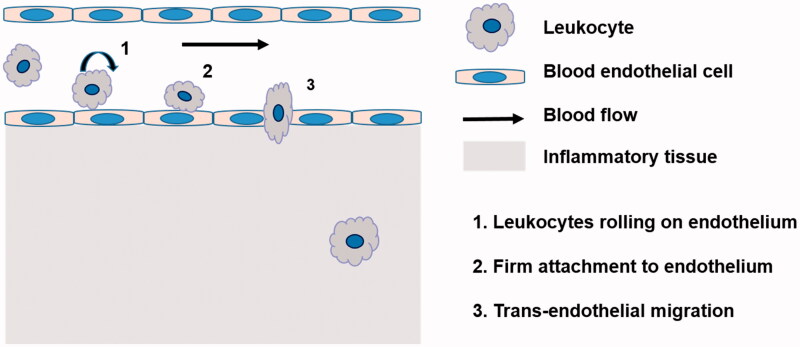
Schematic representation of process of leukocyte transmigration endothelium under inflammation.

**Figure 3.**
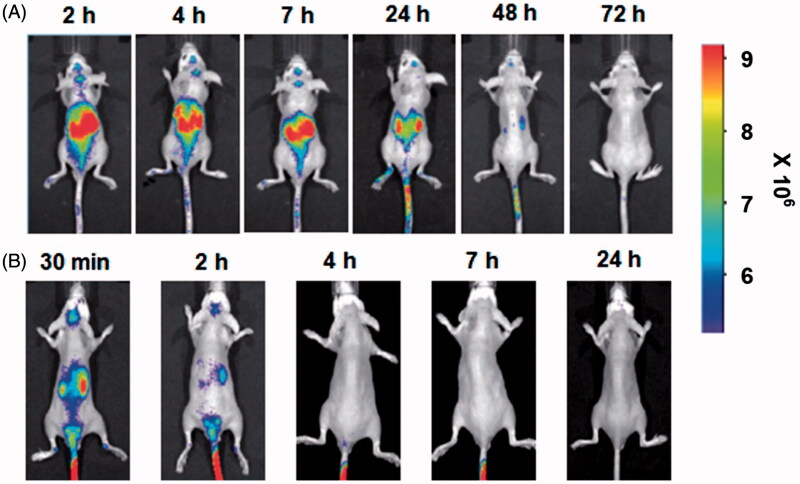
(A) Alexa Fluor 680-labeled BMMs loaded with nanozyme. (B) Alexa Fluor 680-labeled nanozyme administered alone (Brynskikh et al., 2010).

Leukocytes as drug carrier are more attractive in comparison to RBCs. On the one hand, they can act as depots enabling sustained and prolonged drug release, thus prolong the half-life of drugs; On the other hand, leukocytes have inherent ability of homing into inflammation tissues. It is well demonstrated that there is a common inflammatory component in many diseases (Brynskikh et al., 2010; Grivennikov et al., 2010; Lee, 2014). The process of inflammation is characterized by leukocytes mobilizing from the bone marrows or vicinity tissues into blood, and specifically migrating to the diseased sites. When inflammation occurs, the inflammation microenvironments induce the overexpression of the cell adhesion molecules (CAM) on the surface of endothelial cell monolayer, which mediate interactions between leukocytes and endothelial cells, facilitating the initial process of leukocytes rolling, firm attachment to endothelium and transmigration (Penberthy et al., 1997; Wong et al., 2007) (Figure 2). In early studies, microspheres were constructed to mimic the adhesive behavior of leukocytes in response to inflammation by attaching leukocyte adhesive ligands to the surface of microsphere. They owned the property of leukocytes and transmigrated through endothelial barrier into lesion sites (Eniola et al., 2002; Eniola & Hammer, 2003; Omolola Eniola & Hammer, 2005). In the later research, leukocytes were directly utilized as carrier to transport drug. For instance, in 1-methyl-4-phenyl-1,2,3,6-tetrahydropyridine (MPTP)-intoxicated Parkinson's disease model, bone marrow derived macrophages (BMMs) carried catalase NPs, cross the blood–brain barrier and infiltration into diseased tissues. Nanozyme administrated alone was completely cleared from the brain within 2 h, while nanozyme loaded into macrophages was tracked in the brain up to 48 h after administration and showed increased nanozyme accumulation in brain (Brynskikh et al., 2010) (Figure 3).

Rudolf Virchow identified the presence of leukocytes within tumors for the first time in the nineteenth century, which indicated a possible link between inflammation and cancer (Grivennikov et al., 2010). It was reported that inflammatory responses play important roles at all stages of tumor development, including initiation, promotion, invasion and metastasis (Coussens & Werb, 2002; Grivennikov et al., 2010). Hypoxia is a hallmark feature of most solid tumors. Necrotic areas are frequently observed in the center of tumor, resulting from hypoxia. Solid tumors with hypoxic areas have poor prognosis, since low oxygen tumor environments are generally resistant to radiation and chemotherapy, and stimulate tumor progression, angiogenesis and vasculogenesis (Pang et al., 2016). Therefore, these tumor hypoxic areas might be considered to be the candidate target (Lewis & Pollard, 2006; Wilson & Hay, 2011). There is evidence showing that high production of chemoattractants secreted from tumor cells undergoing inflammation and necrosis could recruit a large number of leukocytes, mainly including macrophages and T cells (Steinfeld et al., 2006; Choi et al., 2007).

Based on above understandings, leukocytes as drug carriers into targeted sites have been investigated, particularly in tumor, inflammation and central neural system (CNS) disease. T-cells have been studied extensively for cell therapies for cancer treatment, in early clinical trials, infusing modified T cells have yielded promising results for the treatment of cancer and chronic inflammation. Stephan et al. stably conjugated drug-loaded NPs to the surfaces of therapeutic T cells (Stephan et al., 2010). The *in vivo* tumor eradication potential of NPs-conjugated T cells was also investigated in mice bearing subcutaneous B16F10 tumors. It elicited significant tumor elimination and prolonged the survival of mice. A promising strategy was developed by adopting monocytes as a cellular vehicle for co-delivery of echogenic polymer/C5F12 bubbles and doxorubicin-loaded polymer vesicles toward hypoxia regions of malignant tumors (Huang et al., 2015). Following the intravenous administration, the cell based drug delivery system penetrated to a depth beyond 150 mm from the nearest blood vessels within tumor while NPs entered only 10–15 mm depth.

### Stem cells

Over the last decade, stem cells have emerged as promising therapeutic options for the treatment of many diseases. As with leukocytes, stem cells exhibit an intrinsic tropism to sites of injury, inflammation and tumor (Muller et al., 2006; Porada & Almeida-Porada, 2010). Marrow-isolated adult multilineage inducible (MIAMI) cells, a subpopulation of mesenchymal stem cells (MSCs), were selected as cellular carriers for the delivery of NPs into brain tumors. MIAMI cells loaded with NPs were found to specifically localize between tumor cells and normal brain parenchyma following contralateral injection into mice with U87MG glioma (Roger et al., 2010,2011). The possible mechanisms have been proposed which may contribute to the homing property of cell, the interaction between cell surface receptors and chemokines/cytokines secreted by diseased site mediated migration, adhesion and infiltration of MSCs into the diseased sites (Hu et al., 2010; Bexell et al., 2013).

In the terms of stem cell itself, first, it has the capacity to differentiate into a wide variety of functional cells, which could be used to repopulate and repair the damaged living tissue; second, it can secret trophic factor to nourish the damaged neuron and promote functional recovery (Borlongan et al., 2011; Huang et al., 2012). Most neurodegenerative diseases such as PD, AD, epilepsy and stroke induce a localized loss of neurons. Therefore, stem cells have been widely used in the fields of regenerative medicine and CNS diseases (Peng et al., 2012; Adams et al., 2013; Felsenstein et al., 2014; Yin et al., 2014). After middle cerebral artery occlusion (MCAO), transplanted MSCs tend to migrate into the region of the ischemic hemisphere rather than to the contralateral non-ischemic hemisphere and they have been found to significantly improve neurological functional recovery (Chen et al., 2000).

Despite mounting laboratory results showing that many stem cell populations exhibit a prominent tropism to ischemia, inflammation and tumor, the transfer capacity of stem cells is still being questioned. In order to enhance the targeting capability of stem cells, MSCs engineered to overexpress receptors are an alternative way to enhance MSC migration, since receptor/ligand interaction plays an important role in MSCs homing (Hu et al., 2010). The MSCs transfected with lentiviral vectors (LV) carrying the chemokine receptor four (CXCR4) gene promoted migration to the injured regions due to the overexpression of CXCR4 (Dar et al., 2006). The interaction of chemokine/cytokines secreted by inflamed tissues and receptors attract MSCs homing to inflammation area. Similarly, gene therapeutic benefits can be further expanded in a similar method. Genetically modified MSCs expressing tumor necrosis factor (TNF)-related apoptosis-inducing ligand (TRAIL) exhibited targeted delivery and local production of TRAIL at glioma tumor site (Menon et al., 2009).

Yang et al. have jointly exploited NPs and stem cells to deliver vascular endothelial growth factor (VEGF) into ischemic hindlimbs to enhance angiogenesis (Yang et al., 2010). In fact, the stem cell alone can promote vascularization and tissue regeneration, but the function is limited due to insufficient expression of angiogenic factors. The combinational application of NPs loaded with VEGF and stem cells led to two- to four-fold higher vessel densities two weeks after implantation compared with control cells or cells transfected with VEGF by using lipofectamine 2000. In similar way, NPs act as contrast agent for imaging can label and track transplanted stem cell noninvasively. As reported by Huang’s lab, ^64^Cu labeled hyaluronic acid coated mesoporous silica NPs (HA-MSN-^64^Cu) were constructed and incorporated into MSC. The MSC-NP system overcame blood–brain barrier and actively targeted orthotropic glioma. In order to evaluate the homing capability of NPs with MSCs, HA-MSN-^64^Cu with the same amount of radioactivity was also injected as a control, PET imaging demonstrated that there was a 5.2 ± 1.3-fold higher tumor accumulation in the glioma by MSC-HA-MSN-^64^Cu within 24 h after injection than the free HA-MSN-^64^Cu (Huang et al., 2013).

## Construction of “cell-NPs” drug delivery system

### No-covalent coupling

**Figure 4.**
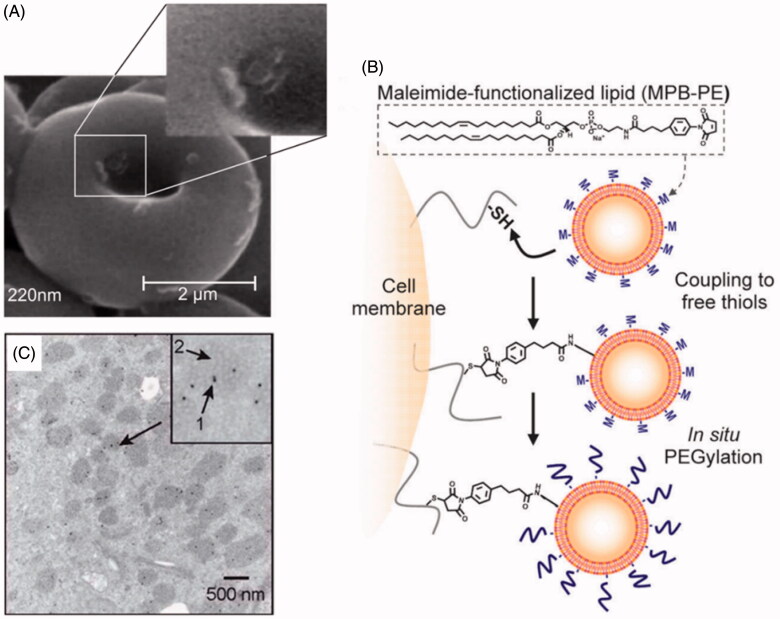
(A) NPs absorbed onto RBCs surface by no-covalent coupling (Chambers & Mitragotri, 2004). (B) Covalently coupled NPs onto T-cell membrane via thiol-maleimide conjugation (Stephan et al., 2010). (C) NPs internalization by macrophages (Choi et al., 2012).

No-covalent coupling just requires simple mixture, then polymeric NPs could be adhered onto the RBCs surface through non-covalent interactions between polymeric materials and the RBCs, such as van der Waals, electrostatic, hydrogen bonding and hydrophobic forces (Villa et al., 2015) (Figure 4A). Particle size and particle/cell ratio are two important factors to decide the number of particles attached, high particle/cell ratio could improve the amount of NPs adhesion to the RBCs surface, but excessive amounts of particles will lead to cell agglutination. Under the same ratio of cell/particle, the amount of particles attached varied with particle diameter (Chambers & Mitragotri, 2004). Optimized particle size and particle/cell ratio are critical during “Cell-NPs” preparation. Anselmo et al. anchored PLGA NPs onto the surface of RBCs by no-covalent coupling to significantly prolong the blood circulation time of NPs, and reduce their uptake by liver and spleen (Anselmo et al., 2013).

The advantages of this method are that adhesion of NPs did not compromise key cellular functions, and no hemolysis of RBCs was observed. However, this no-covalent coupling is too fragile to control the behavior of NPs (Anselmo & Mitragotri, 2014). Upon intravenous administration, particles easily desorbed from cells likely due to blood flow induced shear stress or cell–cell interaction (Chambers & Mitragotri, 2007). It is thus essential using covalent binding to increased adhesion strength.

### Covalent coupling

To stably couple synthetic drug carrier NPs to the surface of therapeutic cells, we exploited the fact that many cells exhibit high levels of functional groups on their surfaces, such as thiol group, amine groups, sialic acid residues, etc. (Chambers & Mitragotri, 2004; Stephan et al., 2010). The level of functional groups varied with cell types. This method demands chemical modification of NPs to facilitate covalent conjugation with cell carrier. Covalent coupling can bound NPs more firmly to the surface of cells, and avoid easy detachment from cells in blood flow (Puentes et al., 2000; Bradley et al., 2002). Take for example, reactive maleimide groups modified NPs could be covalently linked onto the surface of thiol rich T-cells via thiol-maleimide conjugation. With this strategy, a substantial number of NPs with diameters in the 100–300 nm range were conjugated onto T cell surface by a simple two-step process: cells were first incubated with NPs to permit maleimide–thiol coupling, followed by *in situ* PEGylation with thiol-terminated PEG to quench residual reactive groups of particles (Stephan et al., 2010) (Figure 4B). T-cells carrying NPs exhibited unaltered transmigration efficiencies compared to unmodified cells. After crossing the endothelial barrier, T-cells retained 83% (±3%) of their original NP cargo physically attached. However, this tightly covalent coupling will make it difficult for NPs to separate from cell carriers. Therefore, it is necessary to design drug delivery systems that keep stable in circulation and display high NPs detachment from cell under environment stimulus in the following research.

### Internalization

It has also been widely demonstrated that leukocytes were able to efficiently phagocytize NPs by endocytosis (Choi et al., 2012) (Figure 4C), highlighting their advantages over other cells as drug carriers. The “Cell-NPs” drug delivery system can be achieved by simply incubation. Tao et al. showed BMMs can efficiently uptake nearly all the PF-PTX-micelles and PLGA-PTX-NPs within eight hours after incubation at 100 μM, more than 80% of PLGA-PTX-NPs or PF-PTX-micelles were released from BMMs into the extracellular milieu at four days and increased to 90% by five days (Tao et al., 2013).

Receptor mediated internalization requires target moiety modification on the outer layer of NPs (He et al., 2016). The typical example is that RGD was used as the targeting ligand for integrin receptors expressed on neutrophils and monocytes surfaces to facilitate cell uptake of liposomes (Jain et al., 2003; Qin et al., 2007, 2014a,b, 2015). The distribution of RGD-coated magnetic liposomes in the brain was found to be relatively higher in a brain inflammation rat model by orthotopically injecting IL-1β into striatum, and the ratio was 1.5 times of the uncoated liposomes. Moreover, the result of *in vivo* cellular sorting also exhibited monocytes/neutrophils which can uptake more RGD-coated liposome than the uncoated (Jain et al., 2003). A relatively higher coating density of ligand containing liposomes led to an increase in uptake in inflamed cells because of a multivalent effect (Kang et al., 2011; Gao et al., 2013).

Most noteworthy is the work carried out by Afergan et al. on negatively charged liposomes to deliver serotonin to the brain via monocytes (Afergan et al., 2008). This study was designed carefully using double-radiolabeled liposomes, ^3^H-liposomes and ^14^C-serotonin for co-localization imaging, ^3^H to ^14^C ratio normalized to the injected dose had no significant difference in brain, which confirmed that intact liposomes enter into brain tissue.

## Others

**Figure 5.**
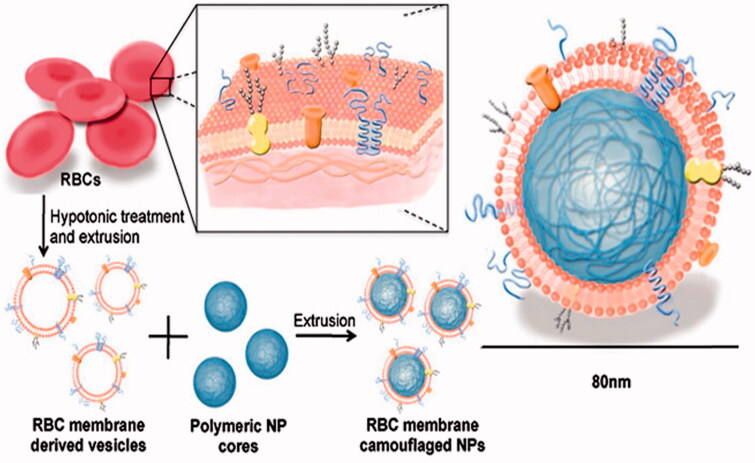
Schematic representation of the preparation process of the RBC-membrane-coated PLGA NPs (Hu et al., 2012).

Currently, other similar strategies have created excitements in the drug delivery field. They appear to have multiple advantages over current synthetic drug delivery systems. NanoPorous Silicon particles (NPS) were coated with cellular membranes purified from white blood cells. These hybrid particles called leukolike vectors (LLV) have similar function with leukocytes, it is able to prevent rapid clearance of phagocytic cells of the immune system; communicate with endothelial cells through receptor–ligand interaction; transport and release a payload across an inflamed reconstructed endothelium (Parodi et al., 2013). Similarly, Zhang et al. developed an RBC-membrane-camouflaged polymeric nanoparticle platform (Hu et al., 2011,2012) (Figure 5), which elegantly united the advantage of natural RBCs and synthetic polymers. It successfully prevented RES system uptake without side effects and exhibited a long circulatory half-life as compared to PEG coating NPs (Luk & Zhang, 2015).

Exosomes are phospholipid bilayer cup-shaped vesicles with a diameter size ranging from 40 to 120 nm and are secreted by many types cell, such as dendritic cells, T- and B-cells, tumor cells, stem cells and macrophage (Denzer et al., 2000; Thery et al., 2009). Exosomes derived from different types of cells with differing lipid and protein composition have different biological effects and specific target to various cell types (Sun et al., 2013). It can act as natural endogenous carriers transporting proteins and genetic materials between cells in body, in turn, change the phenotype of the recipient cells (El Andaloussi et al., 2013; Record et al., 2014). These findings gave rise to the hypothesis that exosomes could be exploited for delivery of exogenous therapeutic agent *in vivo*. Tumor targeting capability was facilitated by engineering the immature dendritic cells (imDCs) to express exosomal associated membrane protein 2b (Lamp2b), which could fuse to αν integrin-specific iRGD peptide (CRGDKGPDC). Purified exosomes from imDCs were loaded with doxorubicin via electroporation, with an encapsulation efficiency of up to 20%. Intravenously injected iRGD-exosomes showed high delivery to αν integrin-positive breast tumor tissues, leading to inhibition of tumor growth (Tian et al., 2014).

## Conclusion

In the past decades, tremendous resources have been focused on the development of modern and novel drug delivery systems for targeted drug delivery to disease sites. This is due to the urgent global need for effective targeted drug delivery. In this review, we treat problems from a physiological and pathological perspective to offer a better insight to the challenges and opportunities associated with drug delivery system. We highlight the advantage of autologous cells as vehicle to deliver drug to disease sites.

Cell-mediated drug delivery has achieved great advancement, nevertheless, there are many problems remaining to be solved. Most studies have been performed only *in vitro* using cell as carriers combined with nanodrugs. There have been relatively few studies undertaken on lab animals, and even less in humans. The safety issue has often been neglected during the lab research stage, so these approaches must be assessed for their safety, risk and benefit for patients. There would be some difficulties with storage and contamination, together with the difficulty in encapsulating substances in autologous cells and the absence of a recognized industrial procedure for preparing these kinds of carrier.
